# Supplementary material for: σ54 (σL) plays a central role in carbon metabolism in the industrially relevant Clostridium beijerinckii
Source: Sci Rep. 2019 May 10;9:7228. doi: 10.1038/s41598-019-43822-2 (PMC6510779; doi:10.1038/s41598-019-43822-2)
Supplement: Supplementary file 1 — Supplementary files [file 41598_2019_43822_MOESM1_ESM.pdf]

σ^{54} (σ^{L}) plays a central role in carbon metabolism in the industrially relevant *Clostridium beijerinckii*

Rémi Hocq[a], Maxime Bouilloux-Lafont[a], Nicolas Lopes Ferreira[a] and François Wasels[a] [1]
[a]IFP Energies nouvelles, 1 et 4 avenue de Bois-Préau, 92852 Rueil-Malmaison, France

[1]Corresponding author: François Wasels (francois.wasels@ifpen.fr)

Supplementary files

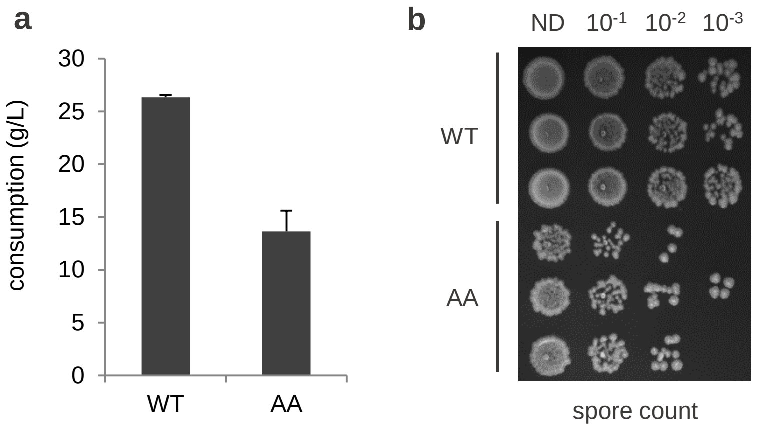

S1 : **Comparative phenotypic analysis of *C. beijerinckii* DSM 6423 wild-type and AA strains, supplementary data.** a. Final glucose consumption measured after 48h of fermentation in Gapes medium for wild-type *C. beijerinckii* DSM 6423 and the AA mutant. Biomass is given as a measure of optical density at 600 nm (OD_{600}). Error bars indicate the standard deviation of triplicate experiments. b. Sporulation assay on 2YTG plates comparing triplicate heat-shocked cultures of the wild-type and AA strains.

Supplementary file S1
(Wasels)

Locus tag	Automatic Annotation	Comment
CIBE_0239	Sigma-54 factor, interaction domain-containing protein	putative operon involved in PP pathway
CIBE_0709	PTS sugar transporter	putative operon involved in fructose/galactitol transport
CIBE_0715	Iditol 2-dehydrogenase	putative operon involved in PP pathway
CIBE_0882	phosphotransferase system (PTS) lichenan-specific enzyme IIA component	putative operon involved in lactose metabolism/transport
CIBE_1109	Phage-rlike protein	
CIBE_1140	chitosanase	
CIBE_1144	PTS lactose transporter subunit IIC	putative PTS transport encoding operon
CIBE_1149	PTS system fructose subfamily IIA component	putative sugar transport/metabolism encoding operon
CIBE_1157	PTS mannose transporter subunit IIA	putative operon involved in mannose metabolism/transport
CIBE_1696	acetoin reductase/2,3-butanediol dehydrogenase	
CIBE_1707	phosphotransferase system (PTS) lichenan-specific enzyme IIA component	putative operon involved in cellobiose metabolism/transport
CIBE_2050	NADPH-dependent butanol dehydrogenase	butanol metabolism
CIBE_2524	Acyl carrier protein	putative operon involved in fatty acid metabolism
CIBE_2531	4-hydroxybutyryl-CoA dehydratase/vinylacetyl-CoA-Delta-isomerase	putative operon involved in 4-hydroxybutyrate conversion to crotonyl-CoA
CIBE_2581	dihydroxyacetone kinase, N-terminal domain	putative operon yielding dihydroxyacetone kinase
CIBE_2622	NADPH-dependent butanol dehydrogenase	butanol metabolism
CIBE_2943	PTS fructose transporter subunit IIA	
CIBE_3081	ketopantoate hydroxymethyltransferase	putative operon involved in coenzyme A metabolism
CIBE_3134	conserved protein of unknown function	
CIBE_3200	cytidine deaminase	
CIBE_3470	NADP-dependent isopropanol dehydrogenase	putative operon, isopropanol metabolism
CIBE_3479	Methyl-accepting chemotaxis protein McpA	
CIBE_3483	ABC transporter substrate-binding protein	putative operon with CoA transferase
CIBE_3485	Citrate transporter	
CIBE_4059	Acetate CoA-transferase YdiF	putative operon
CIBE_4264	putative PTS system fructose subfamily IIA component	putative operon involved in glycosaminoglycan metabolism
CIBE_4670	fragment of fused mannose-specific PTS enzymes: IIA component ; IIB component (part 1)	mannose metabolism
CIBE_4671	putative Transcriptional regulatory protein LevR	
CIBE_5166	Flagellar hook-associated protein FlgK	
CIBE_5246	conserved protein of unknown function	
CIBE_5470	PTS system fructose subfamily IIA component	putative operon involved in fructosamine metabolism
CIBE_5548	phosphotransferase system (PTS) lichenan-specific enzyme IIA component	putative operon involved in sugar transport
CIBE_5598	phosphotransferase system (PTS) lichenan-specific enzyme IIA component	putative operon involved in sugar transport
CIBE_5606	Membrane spanning protein	

PP, pentose phosphate

S2 : **List of *C. beijerinckii* DSM 6423 genes predicted to be controlled by σ^{54}**. Predictions are based on the presence of the σ^{54} consensus motif (TGGCANNNNNNTTGCW) in the correct orientation up to 500 bp upstream of a given coding sequence.

Supplementary file S2
(Wasels)

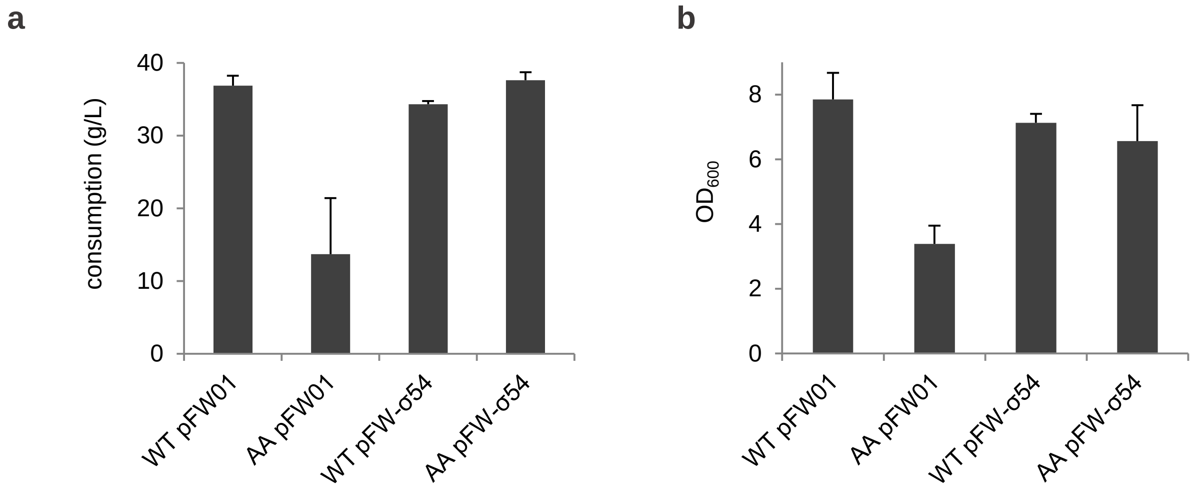

S3 : **Phenotypic analysis of *C. beijerinckii* DSM 6423 σ^{54} complementation in the AA strain, supplementary data 1.** a,b, Final (a) glucose consumption and (b) biomass measured after 48h of fermentation in Gapes medium for wild-type *C. beijerinckii* DSM 6423 (WT) and the AA mutant containing an empty plasmid (pFW01) or the complementation plasmid (pFW01-σ54). Biomass is given as a measure of optical density at 600 nm (OD_{600}). Error bars indicate the standard deviation of triplicate experiments.

Supplementary file S3
(Wasels)

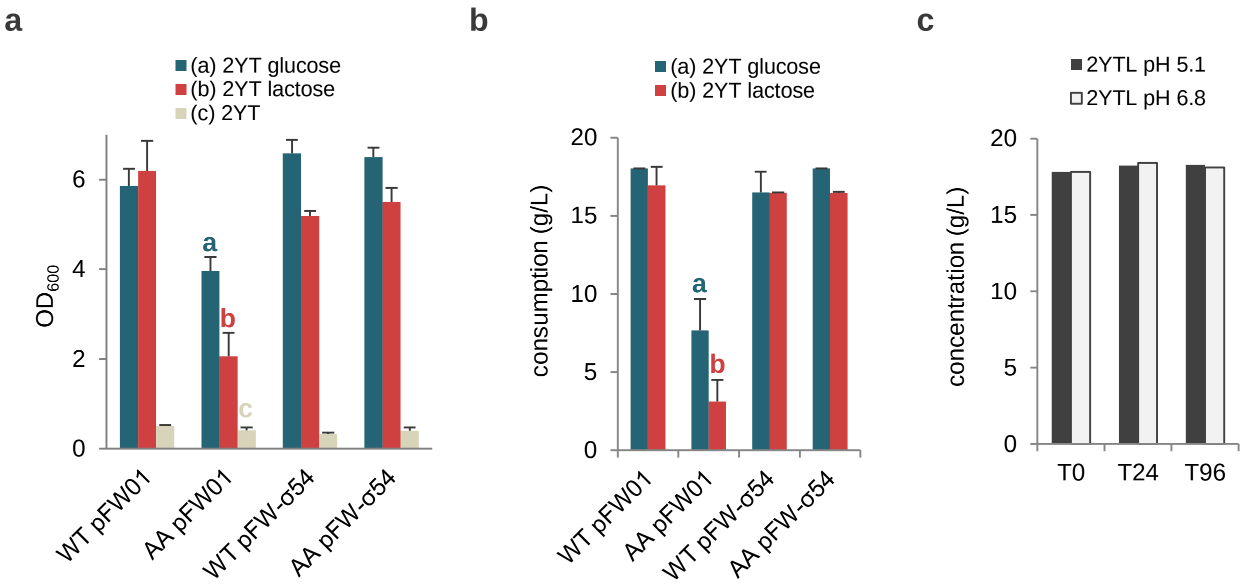

S4: **Phenotypic analysis of *C. beijerinckii* DSM 6423 σ^{54} complementation in the AA strain, supplementary data 2.** a,b, Sugar utilization assay in liquid 2YT with 20 g/L glucose, lactose or without added carbon source. Biomass (optical density at 600 nm, OD_{600}) (a) and carbon consumption (b) are measured 96h after the beginning of the fermentation. c. Lactose does not degrade during a 96-hour period in a 2YT-based medium. Error bars indicate the standard deviation of triplicate experiments.

Supplementary file S4
(Wasels)

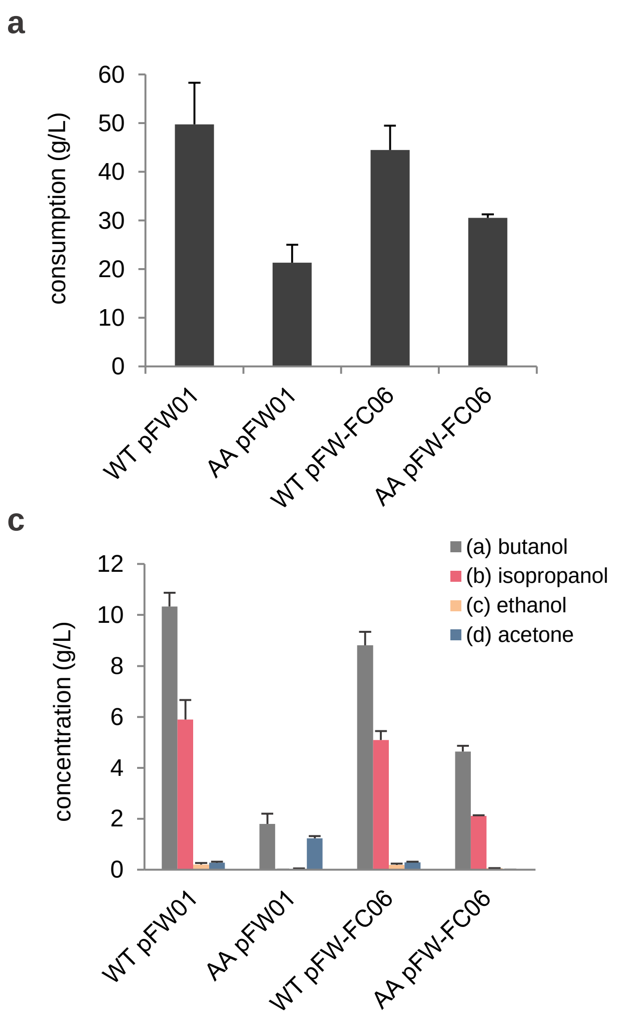

S5: **Comparative fermentation assays in *C. beijerinckii* DSM 6423 wild-type and AA strains overexpressing *s-adh* and *ctfAB*.** a,b,c,d Final (a) glucose consumption, (b) biomass, (c) solvent and (d) acid concentrations measured after 72h of fermentation in Gapes medium for wild-type *C. beijerinckii* DSM 6423 (WT) and the AA mutant containing an empty plasmid (pFW01) or the pFW-FC06 plasmid. Biomass is given as a measure of optical density at 600 nm (OD_{600}). Error bars indicate the standard deviation of triplicate experiments.

Supplementary file S5
(Wasels)

Name	Sequence (5' → 3')
**Construction of pFW-σ54**	
RH077	ACTGTTCTCGAGGACCTGGATGCTGTGGATTAG
RH078	TGACACGTCGACTTATAATCTCTTCCTTGCTGAAGATG
RH086	CAGCAAGGAAGAGATTATAAGTCGACAAAGTATTGTTAAAAATAACTCTG
RH087	AAAAACTAATCCACAGCATCCAGGTCCTCGAGCCGCTTATAATCCATAAC
**Construction of pFW-σ54-AA**	
RH136	TCAATCTTCATGAATTTACCGTAAGTAGAGC
RH137	GCTCTACTTACGGTAAATTCATGAAGATTGA
**Construction of pΔ*sigL***	
RH125	TTGTTATGGATTATAAGCGGCTCGAG
RH126	CAATCTATCACTGATAGGGACTCGAG
guide_sigL_fwd	TCATATTATATTCTGATAATTACA
guide_sigL_rev	AAACTGTAATTATCAGAATATAAT
**Verification of *sigL* deletion**	
RH129	GGTGTTAACTTTATTGAATGTCC
RH130	GCAACTTCTTTTAATGTCAAAGG
**Construction of pFW-FC06**	
MKz01	AAAAAAGGTACCCAAGTATTTTTTATTACATTAATATAGTTAAA
Mkz02	AAAAAAGTCGACTGTTTCATAGTATTTCTTTCT

Target	Name	Sequence (5' → 3')	qPCR efficiency (%)
sigL	0767_F	AGGGTATTTGGAGATATCAACACGA	106,2
	0767_R	GCAATCAACGACTTAACAGATGCT	
gapC	0769_F	AGGGTATTTGGAGATATCAACACGA	104,6
	0769_R	TGGTTCCAATGCCTGTACCA	
adh-1	2050_F	GGAGTAGAACCAGATCCATCAGT	105,2
	2050_R	GTGATCCTCCACCCATAGCAA	
EBP-A	2621_F	GGATGGGACCTTTACAGGAGG	105,3
	2621_R	CGGCATTTCCCCAATTTCGT	
adh-2	2622_F	ACAGTAATGAAAGGCGCAGAAG	106,3
	2622_R	TCCACATAGCCTTTGCAGCA	
s-adh	3470_F	AATTGGCATTGGAGCTGTTGG	105,7
	3470_R	CTCAACACAAATCGGCCTGC	
EBP-B	3471_F	TTGTGGCAATAAACTGTGGCG	105,2
	3471_R	CACCCTCCTCTTTTAGCTCCC	
ald	4606_F	CTCCATATGGCGTTATAGGTGCA	108,1
	4606_R	ACTACAGCATTTCCAGCAGCT	

S6 : **List of primers used in this study.**

Supplementary file S6
(Wasels)

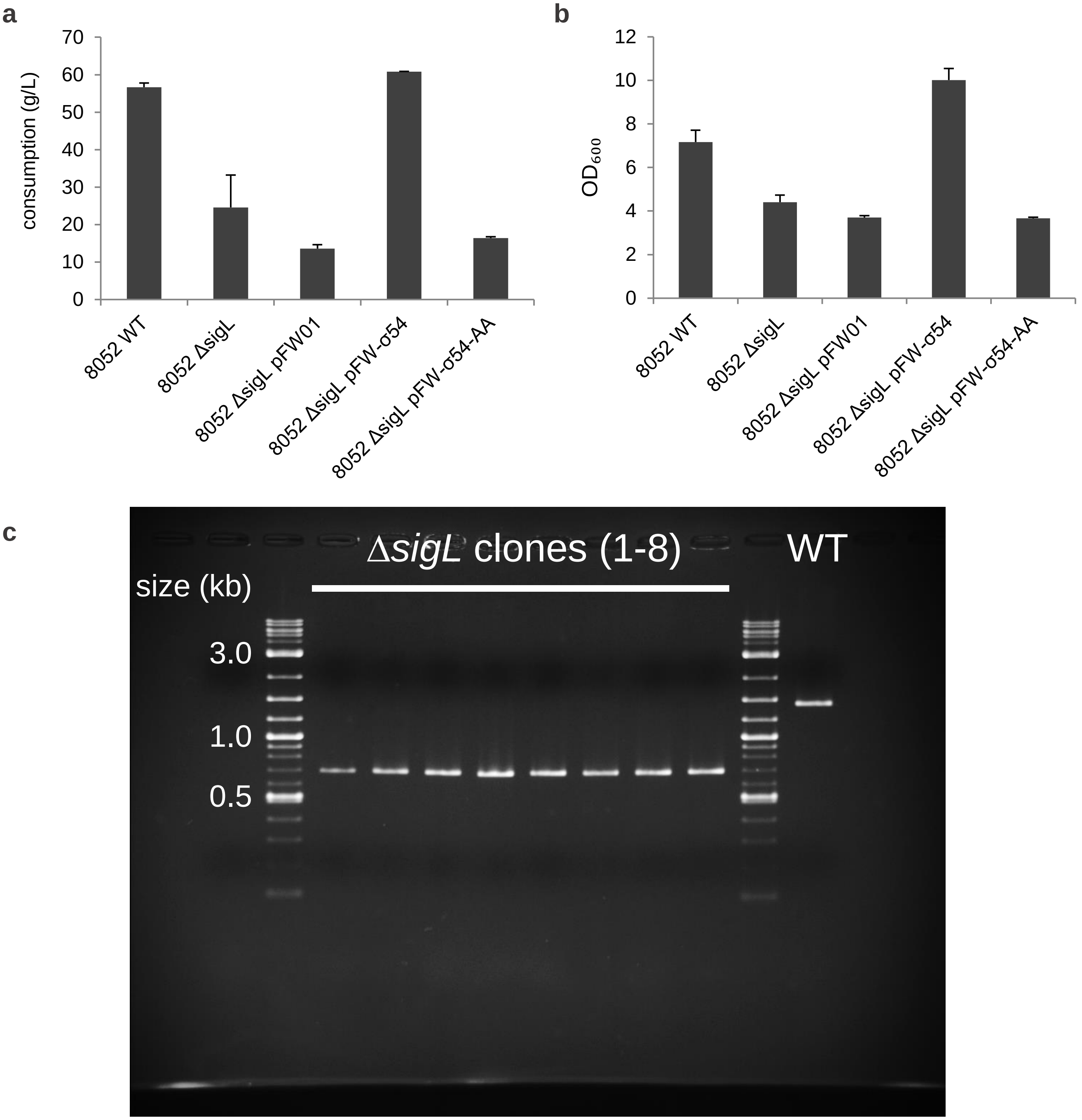

S7: **CRISPR/Cas9-mediated deletion of σ^{54} in *C. beijerinckii* NCIMB 8052, complementation and comparative fermentation assays, supplementary data.** a,b, Final (a) glucose consumption and (b) biomass measured after 72h of fermentation in Gapes medium for wild-type (WT) *C. beijerinckii* NCIMB 8052 and the Δ*sigL* mutant containing an empty plasmid (pFW01), the pFW-σ54 plasmid or the pFW-σ54-AA plasmid. Biomass is given as a measure of optical density at 600 nm (OD_{600}). Error bars indicate the standard deviation of triplicate experiments. c. Verification by colony PCR of *sigL* deletion with primers RH129 and RH130 encompassing the deleted region, full agarose gel corresponding to figure 4b. Expected band sizes are 1406 (wild-type) and 670 (ΔsigL) bp.

Supplementary file S7
(Wasels)
